# Small-world challenges and solutions identified by mid-level managers within a decentralised healthcare system during a qualitative sub-study of a tuberculosis-prevention therapy rollout intervention in Uganda: “When a big drum like the District Health Officer talks”

**DOI:** 10.21203/rs.3.rs-5046392/v1

**Published:** 2025-07-31

**Authors:** Jason Johnson-Peretz, Canice Christian, Cecilia Akatukwasa, Fred Atwine, Moses R Kamya, Diane V Havlir, Gabriel Chamie, Carol S Camlin, Elijah Kakande

**Affiliations:** University of California, San Francisco; University of California, San Francisco; Infectious Diseases Research Collaboration; Infectious Diseases Research Collaboration; Makerere University; University of California, San Francisco; University of California, San Francisco; University of California, San Francisco; Infectious Diseases Research Collaboration

**Keywords:** Decentralization, small world, network, Uganda, healthcare system, tuberculosis

## Abstract

**Background:**

Decentralisation policies that devolve certain administrative and decision-making powers to local levels can pose challenges for public health and healthcare systems. For a decentralised health system to function optimally, mid-level systems must rely on tightly clustered, so-called “small-world” networks to efficiently scale-up national health campaigns and share best practices. Few studies have qualitatively tackled the mechanisms of small-world creation and their potential effects on public health promotion during centralized national campaigns in a decentralised, mid-level healthcare system tier.

**Methods:**

We performed a thematic analysis using a rigorous and accelerated data reduction (RADaR) technique on 23 in-depth interviews and six focus group discussions with mid-level healthcare managers in a cluster-randomised trial from 2019 to 2021, whose intervention component aimed to increase isoniazid preventive therapy (IPT) uptake to prevent tuberculosis among people living with HIV in Uganda.

**Results:**

Training mid-level managers on management and leadership skills fostered the creation of small-world networks within a decentralised healthcare context and promoted mid-level manager agency to address several drawbacks associated with the decentralisation of healthcare systems. Through improved communication, intervention groups encouraged teamwork within their districts, building a denser cluster of networks. This in turn fostered small world ties that paired transparency with a sense of reciprocal accountability moving in multiple directions, upwards to the Ministry of Health (MoH), downwards towards local communities, and horizontally towards peers.

**Conclusions:**

Increased collaboration demonstrably strengthened the clustering of small-world network ties at a horizontal level to disseminate knowledge of best practices more quickly and efficiently in promoting the uptake of IPT while ensuring accountability to peers, the MoH, and local communities, sustaining these levels after a centralized national campaign ended.

**Trial registration:**

NCT03315962. Registered 20 October, 2017

## Background

Decentralisation involves a central government devolving decision-making and other administrative powers “to actors at lower levels in a political-administrative and territorial hierarchy” (1, 2). Beginning in the 1980s and gaining traction into the 1990s, the World Bank and other international organisations promoted decentralisation policies for various sectors in multiple developing countries (3–6). In 1997, Uganda began to decentralise its healthcare system, devolving increased responsibility for healthcare oversight to mid-level health managers throughout the country, which by 2018 comprised 135 districts (7). These mid-level managers included not only District Health Officers (DHOs) tasked with operational planning, health service management, and both HIV and non-HIV care, but also members of the District Health Team such as District Tuberculosis and Leprosy Supervisors (DTLSs), whose responsibilities were more specialised (8). In 2003, to address some of the inherent biases in decentralisation (e.g. variations in financing districts, distribution of medicines, health system performance), the Ugandan government introduced District League Tables with the aim of assessing the range of district performance and improving decision-making quality within the system (7). Nevertheless, as in other countries, decentralisation has tended to bias resources towards urban centres, with rural areas less fully resourced (9–11).

Before being deployed in the health sector, the initial global movement towards decentralisation supposed that decentralisation would break up pre-existing social networks that were contributing to corruption or inefficiency in different sectors (e.g. political, financial), whilst fostering good governance by encouraging democratisation and innovation (5, 11–17). This assumption relied on social network theory, which posits that individuals are linked parts of an interconnected whole, operating interdependently rather than autonomously (18). One type of social network is a *small world*. In a *small world network*, the actors are tightly clustered together, with most actors in the network likewise connected directly to one another with few intermediaries (14). Disrupting the shape of these connexions through breaking up small-world networks and vertically-nested hierarchies into polycentric, localised systems lessens the ability of corruption to flourish by putting greater space between corrupt actors and easing bottlenecks created when all decisions pass through a single actor (19). Breaking up the nestedness of vertical hierarchies, however, sometimes resulted in a consequent loss of horizontal connectivity to mid-level colleagues. As a result, although the fiscal and administrative attention of mid-tier actors became more directed locally, they often lost the ability to share best practices and compare their methods directly with their mid-level peers because information no longer passed quickly and easily between them (4, 5, 16, 18).

Administrative and fiscal decentralisation had the potential to negatively affect health policy goals when health status outcomes became secondary to the concerns of the distinct small worlds of political and economic actors and when the mid-level Ministry of Health administrative units became effectively separated from one another and unable to share knowledge effectively (7, 15, 16). Horizontal connexions between low- and mid-tier personnel could thus become casualties of decentralisation, leading to information asymmetry – a kind of silo effect as mid-level tiers became connected to their neighbours only through a hierarchically-vertical node (3, 12, 13, 20). Mixed results were also evident during attempted scale-ups of national vaccination campaigns, when attempting to focus on the health of populations with high mobility, or when addressing spillover situations like poverty and infectious disease; in each instance, centralised healthcare policies would have been more effective than decentralised ones (5, 16, 19, 21, 22).

“There have always been mixed results from decentralisation in < the health > sector; this is due to the complexity of the process involved and challenges in implementation. Therefore, the desired benefits are not always achieved” (15). Several studies have highlighted challenges which result from poorly or rapidly implemented decentralisation policies (5, 13, 15, 22). Yet to date, most interventions have focused only on the upper levels of the Ministry of Health (MoH), not the mid-level managers (2, 16, 23, 24). Further, although studies have pointed out that mid-level managers often struggle to fit into their role in the wake of decentralisation (4, 9, 12, 17), few, if any, have qualitatively tackled the dynamics among different personnel roles within the same mid-level tier. This limits our understanding of the mechanisms of small-world creation and their potential effects on public health promotion.

For example, in Uganda, DHOs are responsible for the implementation of national programs at the district level. One such programme is Isoniazid Prevention Therapy (IPT) to reduce the incidence of TB disease and stem the transmission of tuberculosis (TB) among people living with HIV (PLWH) (7); TB has been one of the top causes of mortality in people with HIV globally (25–27). In 2015, the WHO, in fact, set a target to reduce TB incidence by 80% and deaths by 90% by 2030 (27). Uganda has historically had a high burden of both TB and HIV (TB: 91.000 persons in 2021; HIV: 1.4 million in 2022); PLHIV made up just over 50% of all TB deaths in Uganda in 2021, despite PLHIV accounting for only 3% of the country’s population (28), and less than 2% of PLHIV were receiving TB preventive therapy by 2018, indicating the need to scale up IPT to curtail preventable deaths.

While earlier implementation questions concerned how to decentralise properly (11, 22), the present question is how to manage emergent, centrally directed health programmes like IPT within a decentralised context. In other words, how can the most effective small-world be fostered in a decentralised healthcare setting? This paper approaches those open questions through an analysis of qualitative data gathered during the SEARCH-IPT study, which incorporated small-world theory to design an intervention targeting middle managers (both DHOs and DTLSs) in rural Uganda and boost provisioning IPT to PLHIV (29). Coincidentally, and due to the need to increase IPT reach among PLHIV in Uganda, this study overlapped with a sudden national scale-up of IPT (30), providing a ‘natural experiment’ showing how targeted training and national scale-up campaigns work differently in the context of a decentralised national healthcare system without intentionally-created small worlds. Intervention sites showed sustained change in IPT initiation after the intervention ended (29), suggesting that by addressing these demonstrated weak spots via the conscious fostering of horizontally connected small-worlds may lead to more sustainable outcomes after centralised, national pushes – like Tuberculosis Preventive Therapy in Uganda – come to an end.

## Methods

### Study setting.

The SEARCH-IPT trial began with 82 Ugandan health districts across three regions in 2017 and 2018, continuing until early 2022. Forty-three districts were cluster-randomised to the intervention, and 39 to a control arm; all managers invited from these eligible districts elected to participate (29). Clusters of 14 districts based on region (east-central, east, and southwest), adjacency, HIV prevalence, and a comparable ratio of urban to rural districts, were randomly assigned by 1:1 pairs to either intervention or control arms at a regional meeting.

While a detailed description of the intervention has been published with the primary results elsewhere (29, 31, 32), two principal elements of the intervention are necessary for understanding this paper: First, the 43 intervention districts received skills-based management and leadership training through biannual mini-collaboratives taught by international business consultants and facilitated by Ugandan experts in TB and HIV from Ugandan research organisations or medical centres, with a primary outcome being IPT initiation rates for adult PLWH over the course of two years (2019–2021); control districts would rely on standard practice. An average of 99% of the 26 eligible managers (both DHOs and DTLSs) attended the biannual meetings for the SW region, whilst the east and east-central regions saw 83% average attendance rate for the 24 and 36 eligible managers, respectively. The mini-collaboratives disseminated knowledge about IPT, and the intervention districts shared data and best practices with their regional cohorts in the course of receiving training in leadership and communication skills. In addition to sharing best practices, participants acquired skills in areas like data-based decision-making, root-cause analysis, quality assurance of data, and self-reliance resulting from implementing their own solutions (33). Small world theory suggests the random links formed at the collaboratives would speed up dissemination of these skills and increase the pace of attitude changes (14). The second element built on this knowledge dissemination by furnishing the DHOs with comparative quarterly progress reports on IPT uptake in all intervention districts, so participants could see how their performance compared to neighbouring districts and generate knowledge sharing opportunities. These collaboratives resultingly fostered the distributed, horizontal small worlds through which information passes.

### Qualitative evaluation.

The qualitative study and writing team consisted of a disciplinarily diverse group of authors based in both Uganda and the United States: medical anthropology (JJP), epidemiology (CC), human geography (CSC), adolescent health (CA), development studies (FA) and clinical medicine (GC, DVH, EK, MK); all are engaged in public health. The Uganda-based authors (CA, FA, EK, MK) confirmed interpretations of the data. This ensured that the translation and interpretation of the data respectfully captured the nuances of participants’ voices. All research was approved by the respective IRBs and entities in Uganda (School of Medicine Research and Ethics Committee at Makerere University School of Medicine (2017 − 116) and Uganda National Council for Science and Technology (HS 233)) and California (University of California, San Francisco (17–22136)).

Over a three-year period (February 2019, January 2020, and September 2021), trained qualitative study researchers conducted six two-hour-long focus group discussions (FGDs) with participant observation immediately after the mini-collaborative meetings to evaluate the impact of the intervention. All DHO and DTLS participants were invited to attend the FGDs. We had one FGD composed of only DTLSs in the East region; the others included both DHOs and DTLSs in the same group. The same researchers conducted in-depth, semi-structured key informant interviews (KIIs), lasting less than 45 minutes on average, with 23 control district key informants at the DHO or DTLS level (out of 77 eligible managers in 39 eligible districts; or approximately 30% of eligible participants) between February and August 2019 and September and December 2020. All participants gave written, informed consent to participate prior to study activities. FGDs and KIIs were conducted in English, and were developed by one of the study authors (CSC).

We did not conduct focus group discussions among control study participants because the control groups remained individualised (per the decentralised model) during the study and therefore functioned as a comparison group that attested to the ongoing, non-intervention conditions within the healthcare system. As described elsewhere, FGDs and KIIs developed for this study followed a similar semi-structured interview format (31, 32). All participants answered questions on the TB situation in their district, patient perspectives on preventative therapy, stakeholder engagement, logistics and resources, clinical policies, and in-district communication with both front-line staff and MoH officials. For the FGDs, perspectives on the intervention content and impact were also topics of discussion. These questions were informed by, but not limited to, small world theory; the questions remained open-ended. We include the guides as supplementary material. Mini-collaborative participants also received a travel reimbursement from their home districts to the mini-collaboration venue; qualitative team members travelled to the control DHO sites to conduct KIIs.

Ugandan study team members recorded and transcribed participant conversations, reviewing them for quality assurance before analysis. The first author, effectively blinded to both the language of the trainings and intended study outcomes in virtue of being brought onto the study after the FGDs and KIIs were conducted, then analyzed the qualitative data using the RADaR (Rigorous and Accelerated Data Reduction) framework analysis technique (34), with the main research question focused on how participants felt about the standard national approach to IPT rollout and their own district-focused leadership skills. The RADaR technique, a systematic and iterative process to identify and condense salient data in the service of a single research question using spreadsheets or word processing software, was chosen because the data set was relatively small and a rapid analysis was necessary for presenting preliminary results to the study leadership within a short turnaround time. Framework analysis after presentation and group feedback further refined the initially identified themes, as the initial reduction uncovered broad thematic groups divisible into context, management, and structure. The ‘structure’ label referenced relations between different levels of the healthcare system or specific roles within it. While the label ‘structure’ was ‘intervention agnostic’, it accounted for agency in the way people work within or around institutional constraints.

Further data reduction identified sub-themes within the ‘structure’ group, which we organised around two main areas of interest: 1) DHO communication challenges, including upwards to MoH and downwards to frontline workers; and 2) DTLS- and TB focal person-reported challenges. Although small-world theory informed the design of the mini-collaboratives, its relevance for addressing some unintended consequences of decentralising polices was only recognised during this final analysis stage when we began to more deeply examine the potential origin of the pain points that fostering small worlds helped overcome. Final analysis of the data thus pursued the research question: Which middle manager-identified pain points (persistent, recurring sources of trouble that cause annoyance) stem from healthcare system decentralisation, and how did intervention participants learn to address these during the SEARCH-IPT trial?

## Results

In-depth interviews with control group participants and focus group discussions with intervention districts revealed structural dynamics that hindered the establishment of inter-district small-world networks. FGD participants reported multiple challenges (‘pain points’) inherent to decentralised healthcare settings, including issues of authority, role clarity, decision-making space, and buy-in; these were especially evident in DTLS and TB focal person roles and the hurdles they faced in executing their responsibilities. DTLS participants reported several challenges, two of which the Ugandan DHOs themselves confirmed: DTLSs lacked authority, yet were responsible for supervision, and they needed to own data, yet in meetings were bypassed by DHOs who went directly to biostatisticians. After laying out these challenges, we then present examples of DHOs learning to use their decision-making space to manoeuvre at the mid-management level and create horizontal ties with peers without the risk of vertically re-centralising the healthcare system, addressing the painpoints highlighted by DTLSs along the way. Finally, we show how trial intervention participants overcame the key obstacle of motivating teamwork in the District Health Team as a whole by strengthening small-world ties through open communication.

### Pain points highlighted by DTLS and TB Focal Person roles.

Although both DHOs and DTLSs are considered mid-level managers, the DTLS role appeared to be the most isolated link at the district level in the small-world network of Uganda’s decentralised healthcare system. A DTLS is, first and foremost, a frontline health worker (e.g., a clinical officer assigned to a health facility in the district) who is assigned an additional administrative role on the District Health Team to oversee TB services. DTLSs therefore sometimes serve as the DHO’s proxy.

Interviews revealed occasional cross-communication over the exact role of the DTLS, potentially compromising the efficacy of the decentralised model to respond to TB. When asked what motivated him to keep implementing TB prevention, one DTLS directly replied, “There’s no benefit a DTLS gets from implementing TB prevention.” Upon deeper analysis, we uncovered three potential areas of misperception around the DTLS role as highlighted by both DHOs and DTLSs. These areas concerned the DTLS role’s authority, data management and ownership, and competing responsibilities, each associated with unintended consequences from decentralisation and overcome through fostering small world networks. The associated position of TB focal person, more often found at the frontline level, is a fourth subtheme emerging in the context of the DTLS managerial role.

Both control and intervention DTLSs indicated limits to their authority, our first subtheme, and both control and intervention participants highlighted the centrality of DHO support for buttressing the authority they did have. Control participants desired DHO accompaniment not only to address this, but also as a way for the DHO to become acquainted with the on-the-ground work performed by the DTLS:

“I am a DTLS. However, the people that I supervise – I do not have absolute power or authority towards them … So, when a big drum like the DHO talks about something people tend to pick it up. …Now that would also give us power as the DTLS: you also stand firm because people will hear you because of your boss.”

DTLS Intervention

This lack of independent authority, or lack of effective ties between DTLSs and the people they supervise, blended into the second subtheme, cross-communication about DTLS roles and data management, as DTLSs reported that DHOs did not always fully appreciate the activities of the DTLS:

“… if I moved with < the DHO>, maybe he would give strength to my visit so that even the people I supervise could feel the importance of it. …Much as I do that work on behalf of the DHO, at least once in a while, he should be moving out with me so that he also appreciates what I report to him.”

DTLS Control

This DTLS suggested that DHOs had developped professional ties with members of the District Health Team in a rather siloed fashion, i.e. one-on-one with the DTLS, one-on-one with the Biostatician, and one-on-one with the DTLS’s supervisees, rather than fostering a closer network of ties between all members of the team. Additionally, lack of experience in the field with the DTLS seems to have fueled and been fueled by divergent expectations between the DTLS and DHO about the DTLS role, extending into the realm of data management. The DTLSs understood the role means being a supervisor who must demonstrate ownership of the data, not only by reporting statistics to multiple team members, but also by doing data cleaning and validation. However, when asked if it was the role of the DTLS to manage the TB data, one control DHO noted:

“They < the DTLSs > are not taking part in the data collection, all they do is to get the data from the biostatistician at the end of the month. For example, for orders of IPT, they are supposed to take part because they should be knowing the stocks and all that, but you realise that they are so not into it. <…>Every DTLS should own the data totally and just approach the biostatistician when they get technical challenges.”

DHO Control

The importance of DTLSs ‘owning’ the data was illustrated by a control-group DHO who could easily bypass the DTLS for data information by going directly to the biostatistician: “I have not done a study to see how many patients go without INH, but I can get that from the Biostat because it is an indicator that is reported on, on a monthly basis.” Yet even when a DTLS challenges the idea that they are mere data-collectors, both control and intervention DTLS participants desire greater recognition of DTLS responsibility for the data, and allocation of time to realise that responsibility:

“I don’t know whether public service is to reinstate the cadre of DTLS into the structure of DHT < District Health Team > so that the DTLS will have time to do the supervision – because it is not data collection as some people sometimes allege. And now that the reporting has been synchronised into the DHIS < District Health Information Software > tool, we would see the DTLS do the supervision, do the validation, and the information will go to the system. And for you to be able to go and support this facility, then you need time.”

DTLS Control

After the intervention collaboratives, intervention participants highlighted an increased recognition by DHOs of this component of the DTLS role. As one DHO succinctly said, “The DTLSs were being trained to improve the management of data.” DHOs in intervention districts subsequently made more effective use of their DTLSs by requesting weekly reports.

“Particularly in my place, we experienced poor capture of TB cases, and as such, I was challenged. Actually, I informed my DTLS to increase the number of engagements we have together with the frontline health workers so that we increase our yield. In so doing, we also considered IPT as part of the core ingredients of the communication to be passed on to the providers.”

DHO Intervention

Greater confidence in the ability of the DTLS to manage data also led to a consequent increase in decision-making based on that data, especially in tracking facilities which were not initiating IPT. Giving the DTLS the responsibility for reporting to the entire district health team, with the DHO present, gave the DTLS a more visible role and more authoritative voice, while fostering the intra-team horizontal ties necessary for effective rollout and scale up of IPT in the district.

Data management, however, remained only one of several competing responsibilities for the DTLS. This is especially evident during staff turnover:

“Now you have 17 facilities to supervise. So you will just supervise a few and make a report out of those, but you can’t cover all of them because you are also needed in your facility. And also we are understaffed again; I am the only clinical officer in my facility and I am the in-charge.”

DTLS Control

This was common to intervention and control districts, who responded similarly in giving the DTLS time to be in facility and in the field:

“We designated the DTLS to have some time so that he can be able to do follow-up in individual health facilities and mentor some new health workers, and then we have redistribution of drugs taking place in our region.”

DHO Intervention

“The support I get helps me to move out, and then also the district gave me some days that I am not supposed to be at the facility – that is when I am supposed to do district work. Those are two days in the week, so that motivates me so much.”

DTLS Control

Importantly, the difference in these two approaches lay more in the intervention group giving time for the DTLS to mentor new health workers, an aspect of the role neglected in control districts, yet essential in creating small world networks. In contrast, despite extra time, a control group DTLS implied he did not have the skills to budget time or create timelines, compromising his responsibilities to Implementing Partners. Overwork and a lack of capacity weakened leadership efficacy not only for the DTLS but, by extension, for the DHO as well.

When DHOs recognised that TB is not a one-person job, they sometimes brought on a subdistrict TB focal person, our final subtheme in this section. (35) One intervention district used specific lessons from the mini-collaborative, building broader buy-in for their vision by engaging people with positive outlooks to build up interest in managing TB:

“In our facility, I had mentored somebody to be like the health subdistrict focal person for TB, but this person went away for school … But when we had a meeting, I talked about a gap which was there and said we need people to come and support TB management. The only statement I made was that ‘whoever is willing to come and work with me in TB, I am ready to train you.’ And then one person started by showing interest. … and many people now started coming in; they want to learn how to manage TB patients. So, I want to encourage my colleagues that working with those who are positive will also change the < previously indifferent > attitude of those other people and they will begin coming in.”

DTLS Intervention

The subdistrict TB focal person in our intervention case presented an opportunity not only to alleviate the overwork of the DTLS, but also to build interest in TB prevention more broadly among the team. By definition, though, a ‘one-man issue’ does not make a small world; creating a small world meant bringing others on board by strengthening ties between district and sub-district levels. This seemed to be a unique case among our intervention groups, but was shared with other managers during the FGD. In fact, the intervention groups were explicit about the give-and-take nature of communication with and data management by their TB focal people, highlighting the structural links that role needed in order to function well:

“We tasked the district focal person to keep updating the team, the frontline health workers on the performance of each health facility that is participating. So, what he does, he picks up a phone and calls to find out how many have been initiated, and then he summarises that … and shares < it > on the platform of the health workers.”

DHO Intervention

The focal person thus coordinated and ‘centralised’ data on behalf of all participants in the district. Control districts, however, seemed not to fully engage the possibilities of such a role, not really delegating responsibility for the coordination of data to a focal person, though allowing them to report their challenges to the team. For the DTLS participants, the multitude of responsibilities, lack of time management training, and cross-communication with their DHO all combined to create a frustrating, but addressable dynamic amenable to multiple solutions once different districts began to communicate with one another horizontally.

### Agency and the creation of small world ties within a decentralised healthcare system.

Like with the DTLS role, a similar challenge in decentralised healthcare systems is a lack of clarity on the extent of the DHO’s power to expand and interpret policy (36). Overall, participants recognised that the Ugandan healthcare system needs multiple network connexions to function effectively:

“Perhaps at the time when someone was writing this project down, someone looked at the DHOs and considered them key players in service delivery. But you see there are many pieces that have to be moved for services to be delivered. … you have pushed the piece for the DHOs’ motivation, information … but there are other pieces that must be pushed. We have talked about the facility in-charges at the lower health units; that is the piece that must be pushed. Individual pieces – for some of you who know the game of chess – there are castles and bishops and queens that must also be pushed at the ministry level and inter-ministerial level because that is where the majority of the decisions are made.”

DHO Intervention

Coordinating those moving parts, especially when they function at the same mid-tier management level, presented challenges in the context of a decentralised healthcare system. Among our participants, control districts seemed reluctant to recognise themselves as actors forming a vertical link between policy makers and policy implementers, or the “people on the ground” plus the local communities whom they served. This compromised both the morals and morale of control-site DHOs when the MOH indicated IPT roll-out to Health Centre IVs (fourth-level health centres) and said nothing about smaller clinics. As control sites reported, their effectiveness in executing these responsibilities was sometimes hampered by a very real lack of resources, including fuel, stationary, air time for phones, and even a computer in the DHO office. In such a context, one control DHO expressed reluctance to creatively implement policy around increasing IPT uptake, citing “the public standing orders section 9, ‘what is not provided, you cannot authorise’” (*DHO Control*). In contrast, the intervention groups became more dynamic when faced with resource constraints:

“We even go beyond where the research study is. I even went beyond where the < Implementing Partners > wanted: they had sampled some health facilities, but for me, I said, ‘I will go everywhere ART is being provided.’”

DHO Intervention

Intervention sites suggested the reason they began to exercise their authority more effectively was that they had learned more about their own position in relation to the MoH nationally and to the communities they served locally. In one intervention-group DHO’s words, “the missing link was the organisation and implementation of IPT, the linkage between the man on the ground and the person who is making the policies.” This recognition is exactly the awareness needed to consciously create the multi-directional ties that lead to small world networks and foster a sense of accountability that doesn’t look solely upwards to hierarchical superiors (e.g. MoH officials). Importantly, this empowerment that the intervention group expressed around connecting the policymakers with the people on the ground also touches on the moral fibre of the DHOs, and thus also on their morale. As one control informant noted when raising an ethical question regarding distribution of INH,

“You remember when we were talking about Isoniazid, that we started implementing it at only Health Center IVs? But diagnostic centers go beyond Health Center IVs. Are we unfair to clients who cannot access Health Center IVs? So, while the policy is okay with it, it has some implications – and that is discrimination.”

DHO Control

Health centre IVs, being larger than clinics are generally located in more populous or central areas, but district leaders felt accountable to the people even from the smaller or more distant communities, too. The intervention group, in contrast to the control group, felt empowered to address that ethical dilemma using the tools they learned through the intervention, without feeling that their team’s agency was going against or beyond policy guidelines.

In contrast to control districts, intervention DHOs gained a sense of ‘implementation confidence’ by improving communication, making presentations to and getting feedback and support from their colleagues, and by subsequently implementing self-initiated sensitivity training for frontline workers. As one intervention arm DTLS put it, “We have shared a variety of challenges and we have also shared experiences on how to overcome such challenges.” Contrasting the SEARCH-IPT mini-collaborations with MOH meetings, one DHO observed,

“I told < a colleague > that your < SEARCH-IPT trial > meetings are more engaging and they are task-oriented because in them you ask many questions, for instance: how, where, and what – among other questions. So in summary, your meetings are engaging, task-oriented, and informative – especially from the presentations that are usually given when the team meets.”

DHO Intervention

The horizontal give-and-take of the SEARCH collaboratives was more productive for innovative policy implementation than a strictly top-down approach had been. The ability of the DHO to create that link with ‘the person-on-the-ground’ seemed compromised when DHOs felt national directives constrained the decision-making space locally. The back-and-forth questioning of fellow DHOs, and especially (mentioned by several participants) the expectation that they will present to their fellows, gave the DHO an increased sense of efficacy, a sense of one’s actions generating concrete results and affecting others, in the way that learning-by-teaching often does. Through this sort of collaborative approach, the top-down directive widened out at the district level to foster horizontal ties, facilitating greater engagement with local teams and creating the small worlds that decentralising policymakers may have assumed would automatically be present.

### Small worlds create communication and accountability links within decentralised settings.

Both intervention and control DHOs focused on how open communication was closely tied to a sense of their own power, whether in the face of MOH meetings where they were often passive recipients of knowledge, or (especially for control districts) in terms of uncertainty about the extent of their power to interpret the implementation of those policies. A repeated concern among both control and intervention group DHOs was getting buy-in from frontline staff. While sometimes expressed as ‘downward communication’, participants also framed it as the presence or absence of teamwork among various stakeholders within the district healthcare system; the links of accountability and communication simply were not as strong in an overall decentralised context. Yet the value of teamwork and its relation to morale did not disappear under such conditions, and one control DHO mentioned how seeing things change through teamwork was motivating. When teamwork was missing, however, participants referred to the root issue as an ‘attitude problem’ without a clear solution. The intervention group, though, showed itself able to address teamwork deficits by passing on the lessons they had learned in the mini-collaboratives:

“The most important thing that we did was to get the facility in-charges on board. The special thing that we did was that after our one-day MBA – before we forgot the concepts of customer care, the concepts of quality, and all that – we engaged the facility in-charges and TB focal person. So for us, we got a ‘one-day MBA’ <i.e., the leadership and management training component of the SEARCH-IPT intervention > and we gave them a 3-hour MBA. And it made a difference by the way, because by the time they left, they were all charged up.”

DHO Intervention

These sites effectively multiplied the type of links between the different members of their teams. Such multiplication of ties, however, was not limited simply to the district team itself: other sites brought on Implementing Partners to help mentor staff and sensitise them to the importance of IPT. Unsurprisingly, when DHOs became more accustomed to presenting ‘horizontally’ to their peers as fostered by the intervention collaborative meetings, those skills also extended in the other direction, improving communication between the DHO and the frontline health workers:

“From the time this project came in up to today, I see a downward communication; that is from the office of the DHO to the frontline health workers, and < it > did not only stop there. It actually went down to the sub-county health workers, and these are people who are very active in contact tracing. That is a reason as to why we are seeing an increase in the IPT uptake. Also, at various points of service delivery, we see the same concern: There is increased health talk on IPT, and also contact.”

DHO Intervention

Through improved communication, intervention groups encouraged teamwork within their districts, building a denser cluster of networks. A perceptual shift fostered this improvement by no longer attributing personnel attitudes to self-generating apathy, but instead viewing such attitudes as an opportunity for training and sensitisation.

Ultimately, DHOs in the intervention group collaboratively opened up their own decision-making space, while bringing others into the decision-making process. The intervention thus gave district-level managers the confidence to roll out IPT more widely, while providing the tools to create buy-in and motivate their front-line and district teams. This in turn fostered the small world ties that paired transparency with a reciprocal movement in both directions between actors in their network, in contrast to the unidirectional ties they felt with MOH officials, ensuring that the original purposes of decentralisation could be realized.

## Discussion

This qualitative study, embedded within an intervention trial that applied small-world theory targeting health-system middle managers in rural Uganda, revealed unintended negative consequences stemming from decentralisation and how the active cultivation of distributed, horizontal networks through mini-collaborative meetings helped solve those issues. Despite the many benefits of decentralisation, rolling out or scaling up healthcare policies in countries with decentralised healthcare systems means engaging with the particular structural biases inherent to decentralisation, including issues of upward and downward accountability (4, 5, 13, 22), variability in data quality (15, 20, 24, 35), and the existence of narrow or unknown decision-making spaces – decision-making space being the ability to interpret national policies to suit a local context (9, 11, 17). Reports from Asia similarly identified a lack of capacity development in Nepal after decentralisation, while in Vietnam, role confusion caused decentralisation of the health system to fail (12).

Other studies note that decentralisation creates a two-levered machine with power in the hands of the government as well as the populace. “The big challenge lies in the effort to reconcile these two movements towards common objectives shared collectively” (5). From the perspective of our DHO participants, the key problem was a missing link between policymakers and the ‘person-on-the-ground’. Through the mini-collaborative meetings, intervention groups came to realise themselves as the actors who must forge those links and tighten the bonds between smaller clinics and larger district centres, in both horizontal and vertical directions (15, 16). This finding is consistent with earlier research in both Uganda and Ethiopia among DHOs in rural districts, showing that the relationship between DHOs and front-line facilities was key to ensuring high performance at the front-line level (9, 37). Other studies have stressed the need to strengthen health systems in coordination with each part; scale-up is possible with system-wide coordination even within low-resource and decentralised settings (24, 38).

In this regard, Mansour (2022) recommends that “the transfer of management authority to the local levels should be accompanied by adequate decision-making and management capacities for the health managers to perform better and improve health services,” while Bossert notes that decision space can be widened when DHOs ‘learn by doing’, which is what the mini-collaboratives were intended to encourage (9, 11, 17, 29, 39, 40). Such action-oriented training has been shown to improve outcomes in other studies (12, 23, 37, 41). In our study, through giving presentations to their peers, intervention groups began to form a culture of leadership development that further strengthened the healthcare system, ensuring that ties operated both horizontally and vertically in both directions rather than merely top-down, enabling the greater participation that is one aim of decentralisation (16, 17, 22).

As mentioned in the background section, decentralisation has tended to bias resources towards urban centres, with rural areas less fully resourced. In terms of small worlds, urban areas present multiple opportunities to form these ties by chance encounters on the street or at local entertainment venues, given the developed infrastructure and concentration of resources that foster a greater density of ties between professional networks in cities. Rural areas, in contrast, appear to require more conscious opportunities to share insights, challenges, and encouragement; this may sometimes be a result of simple geographic challenges (31).

## Limitations and future directions

Focused strictly on rural Uganda, this study’s findings may be of limited generalizability. Even within Uganda, since we included only 61% of the total number of districts, our findings may not be representative of urban areas, or districts located in the northern region. Nor did we set out to identify what challenges the DTLSs specifically faced; rather, we intended to examine the effects of the intervention on improving communication between mid-level managers and their teams. However, participants willingly conceded their concerns. In fact, the focus group discussion format with the intervention groups, especially the one with DTLSs alone, may have provided DTLS participants with the solidarity necessary to speak frankly about their shared experiences, even though such social contexts introduce the possibility of a social desirability bias (i.e. only talking about positive perceptions related to the intervention). This solidarity may in turn have resulted from increased trust from the collaborative meetings in which they had been taking part with their colleagues from other districts, and contrasts with the limitations of the individual control group KIIs.

Finally, on a theoretical level, small-world theory potentially, though not necessarily, ignores different challenges resulting from geographic context (i.e. urban, peri-urban, rural) and terrain, and how cultures of network building may differ in those three geographic zones. It may also overlook what facilitates or constrains links from crossing these geographic divides. Future research might take these gaps into account, especially for urban/rural spillover situations and political arrangements. Similarly, although the study saw sustained changes in IPT initiation rates after the intervention ended (29), suggesting that efforts to create small worlds need not entail large expenditures of money or time to be effective (rather, they require intention and sharing of best practices), we do not know for how long those small world ties continue to exist. A longitudinal study, therefore, presents an opportunity for future research.

### Recommendations.

Previous studies have pointed to Uganda’s strong performance in decision-making with respect to managing contracts with private providers, implementing partners, and the construction of district hospitals (9, 11, 17). Such performance relies on individual skills which can be taught, learned, shared, and improved (16, 23). Researchers have noted, however, that mid-level managers are often caught between top-down and bottom-up accountability to the MoH on the one hand, and local communities on the other (4, 13, 24, 42). We suggest moving beyond the upward and downward accountability that researchers in those studies advocate, to include a third dimension: *horizontal accountability* (13, 16). For a small world to form, links between actors must be actively fostered and developed (15, 16, 37, 38, 43). In order to root out corruption and realise efficiency gains through decentralisation (as the World Bank desires), it is not enough to break up the old and hidden small worlds (3, 5, 22). Rather, formerly hidden small worlds must be made transparent to collaboratively express peer norms and decision-making space; this is the role of horizontal accountability and a key lesson intervention participants learned, as we have described in more detail elsewhere (33). Collaborative opening-up of decision-making space, as exemplified in the Rwandan pre-colonial concept of *imihigo*, links the vertical accountability with the horizontal (22).

Although our particpants created a simple solution to the competing challenges faced by the DTLS role by bringing on a subdistrict TB focal person, which matches the findings of other studies conducted in rural East Africa (35, 44), a study in Eastern Uganda described how TB focal persons expressed a need for orientation to their jobs and frustration at being bypassed in decision-making (35). That experience was also reported by mid-level managers in coastal hospitals in Kenya (41) and by our DTLS participants. This indicates a need to address the underlying challenges first so that they are not replicated when TB focal persons come into their roles.

To excel in the Ugandan health system context, a central change would be to encourage DHOs and DTLSs to develop horizontal accountability by sharing lessons and best practices with same-level colleagues in a collaborative, though not re-centralised context as an essential part of refining and assimilating knowledge among professions in high-stakes environments (16, 41, 45). Similar models have already shown some success in Yogyakarta, Indonesia (20). Incorporating short modules like the SEARCH-IPT mini-collaborative trainings into overall health education may also produce good results by preparing practitioners entering the field to engage in ongoing collaborative models with their colleagues (23, 35, 46).

Second, greater clarity around the respective responsibilities and potential contribution is needed for both DHOs and the DTLSs (4, 9). Visible DHO support of DTLS recommendations, either by accompaniment or by giving the DTLS a reporting role in district meetings, helps lend the DTLS needed authority. Giving the DTLS time outside the facility allows them to conduct the legwork necessary for robust data collection and reporting to the DHO, and helps the DHO make more data-informed decisions for the district, but the DTLS must also be trained in timeline planning and management skills to make best use of this opportunity.

## Conclusion

An intervention which increased collaboration demonstrably strengthened the clustering of small-world network ties at a horizontal level to disseminate knowledge of best practices more quickly and efficiently in promoting the uptake of IPT while ensuring accountability to peers, the MoH, and local communities. Participants perceived that identifying pain points and working creatively to solve those challenges led to greater teamwork in reaching collectively set goals within a decentralised healthcare setting. In so doing, the intervention participants ultimately connected policymakers with the people on the ground: frontline staff who implement policy.

## Supplementary Material

Supplementary Files

This is a list of supplementary files associated with this preprint. Click to download.

- SEARCHIPTDHODTLSFGDKIIguides11Jan2021.docx

## Data Availability

Data is available upon request, but is not publically available due to confidentiality concerns.

## Abbreviations

DHO: District Health Officer(s)
DHT: District Health Team(s)
DTLS: District Tuberculosis and Leprosy Supervisor(s)
HC IV or Health Centre IV: Health Centre, Level Four. (For reference, Health Centre IIs are outpatient clinics staffed by nurses; Health Centre IVs are more like small hospitals with the ability to admit patients, carry out emergency operations, and are overseen by senior medical officers.)
HIV: Human Immunodeficiency Virus
IP: Implementing Partner
IPT: Isoniazid Prevention Therapy
MoH: Ministry of Health
PWH: People living with HIV
TB: Tuberculosis
